# The impact of active aging on depressive symptoms and life satisfaction in later life: the mediating role of hopelessness

**DOI:** 10.3389/fpsyg.2026.1796159

**Published:** 2026-05-19

**Authors:** Myung Hyun Cho

**Affiliations:** BK21 FOUR R&E Center for Psychology, Korea University, Seoul, Republic of Korea

**Keywords:** active aging, hopelessness, depressive symptoms, life satisfaction, mediation

## Abstract

**Objectives:**

This study examined whether leading an active life in old age contributes to better mental health, specifically by reducing depressive symptoms and increasing life satisfaction, and whether hopelessness mediates this relationship.

**Method:**

An online survey was conducted among 211 older adults aged 65–85 years in South Korea to measure active aging, hopelessness, depressive symptoms, and life satisfaction.

**Results:**

Older adults with more active lives reported lower levels of depressive symptoms and higher levels of life satisfaction. Mediation analysis further showed that hopelessness was a key mechanism explaining the links between an active lifestyle and both depressive symptoms and life satisfaction. In other words, maintaining an active life appears to be associated with lower hopelessness, which is in turn related to better mental health.

**Conclusion:**

This study underscores the critical role of active aging in promoting psychological well being in old age and highlights the importance of fostering a hopeful perspective on life.

## 1 Introduction

As society ages, promoting mental health and quality of life in older adults has become a pressing issue. Disengagement theory suggests that individuals withdraw from social roles with age (Shanas, 1962), and this, combined with physical decline, retirement, and reduced interaction, can weaken identity and increase psychological vulnerability. Such transitions are associated with a higher risk of depression and lower life satisfaction (St John and Montgomery, 2010; Soysal et al., 2017). These life-course transitions call for greater attention, as progressive disengagement across various domains of life may lead individuals to develop pessimistic views about the future. Disruptions in social identity can further evoke anxiety, which may ultimately extend to existential doubts about the meaning of life. Therefore, addressing hopelessness in later life is critical. However, aging does not inevitably lead to decline; continued engagement in meaningful activities can help maintain well being and identity (Havighurst, 1961). The World Health Organization (2002) emphasizes the importance of “active aging” in maintaining high quality of life and well being. Active aging is a multidimensional concept encompassing three key pillars: Participation, Health, and Security. These dimensions can be understood as reflecting the behavioral, physical, and environmental domains of later life, respectively. In a broad sense, active aging can be viewed as a harmonious balance among these domains. However, to emphasize the intentional and proactive efforts required in later life, the behavioral domain requires more focused attention. Accordingly, this study operationally defines active aging through the lens of Participation. Supporting this focus, Walker (2006) described active aging as a pragmatic concept, asserting that engaging in diverse activities promotes health, which in turn supports economic and social security. Participation in later-life activities has been shown to promote emotional stability, enhance self-worth, and reduce depression, whereas inactivity is linked to poorer psychological outcomes (Armstrong and Oomen-Early, 2009; Haggard and Williams, 1991; Lee and Robbins, 1998; Na, 2004). Thus, promoting an active lifestyle among older adults is crucial for enhancing both psychological and overall life satisfaction.

Research has consistently suggested that maintaining an active life in old age contributes positively to mental health, particularly in areas such as depressive symptoms and life satisfaction. Studies have shown that older adults who engage in activities such as group participation, socializing with friends, and continued employment tend to report significantly lower levels of depressive symptoms (Galli et al., 2016). Similarly, individuals who engage in meaningful social interactions, self-development, volunteer work, and leisure activities tend to report reduced depression, largely due to lower affect variability throughout the day (Cho and Kim, 2023). Moreover, frequent interactions with children, neighbors, and other close individuals are associated with improved emotional well being and reduced depressive symptoms (Kahneman and Krueger, 2006; Litwin, 2000; Wang et al., 2020). All these evidences highlight that active aging plays a critical role in alleviating emotional difficulties, such as depressive symptoms, and protecting mental health.

Mental health in old age can also be understood in terms of hopelessness, defined as negative expectations about one's future, which is generally associated with increased depression and anxiety (Abramson et al., 1989) and decreased life satisfaction (Yildirim et al., 2023). Related research has shown that hopelessness is positively associated with depression and negatively associated with life satisfaction. Specifically, hopelessness predicts greater future depression severity (Alford et al., 1995), greater difficulty in identifying and communicating emotions (Serafini et al., 2020), and reduced positive affect, which in turn lowers overall life satisfaction. Conversely, higher levels of hope are associated with lower depression and anxiety (Abramson et al., 1989; Chan et al., 2024; Corrigan and Schutte, 2023), and with reduced depression even among older adults with significant functional impairments (Hirsch et al., 2011). Similarly, higher levels of hope are associated with greater psychological well being, including positive emotions, life satisfaction, optimism, and purpose in life, as well as better physical health and lower psychological distress (Long et al., 2020; Murphy, 2023; Santo and Daniel, 2018; Snyder, 2002). In other words, a lack of hope can make daily life more difficult, affecting both depressive symptoms and overall life satisfaction.

Hopelessness in old age can be mitigated through active life. Participation in various activities and interpersonal interactions fosters positive expectations and broadens future perspectives. Although empirical studies on the direct relationship between active aging and hopelessness are limited, existing research increasingly supports the protective roles of active life. Zhang et al. (2018) found that cognitively stimulating activities (e.g., reading and playing games) and socially integrated behaviors (e.g., visiting friends, exercising, and attending social gatherings) were associated with lower levels of hopelessness. Additionally, Eraslan-Capan (2016) found that individuals with higher perceived social integration reported more optimistic future perspectives. Similarly, strong interpersonal support networks reduce hopelessness and promote emotional well being (Asghar and Iqbal, 2021; Beedie and Kennedy, 2002). These findings suggest that active engagement enhances older adults' sense of vitality and fosters a more hopeful outlook.

**Figure 1:**
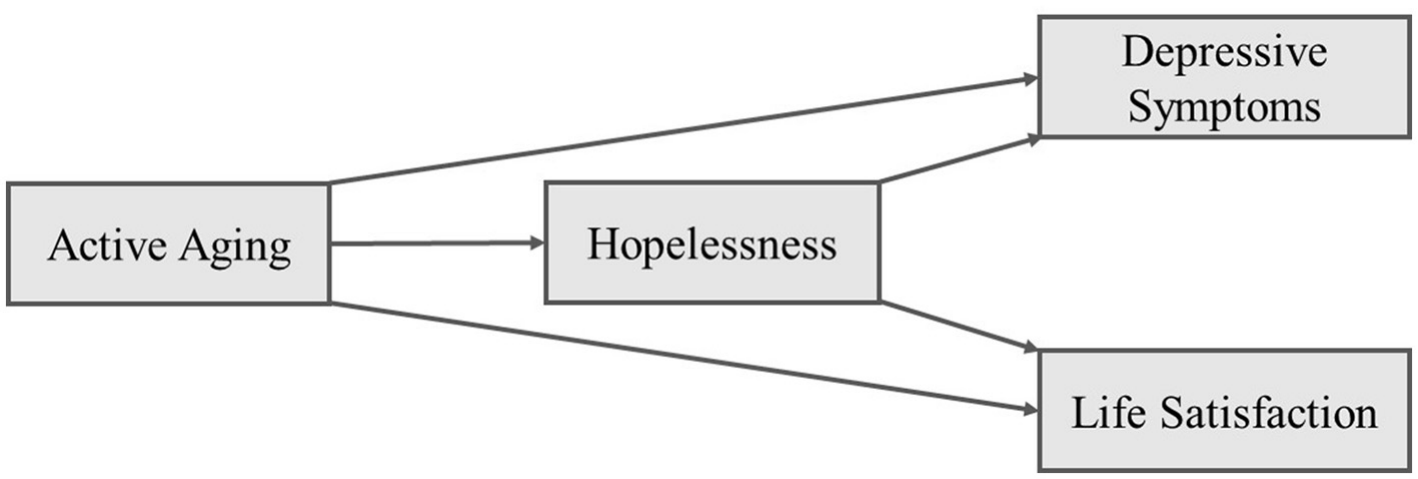
Research model.

Based on this theoretical background, this study examines whether active aging influences depressive symptoms and life satisfaction through its association with hopelessness. First, this study verified whether active aging is linked to lower depressive symptoms and higher life satisfaction, building on previous research. Second, this study tested whether hopelessness mediates the association between active aging and depressive symptoms, given that depression in older adults often stems from a loss of hope. Third, this study explored whether hopelessness mediates the relationship between active aging and life satisfaction, as intentional activity may strengthen identity and foster a hopeful outlook. Overall, this study suggests that active aging is associated with lower depressive symptoms and higher life satisfaction in later life, with hopelessness serving as a mediating factor. The main hypotheses and research model are as follows (see Figure 1):

*H1*: Active aging is negatively associated with depressive symptoms.
*H2*: Active aging is positively associated with life satisfaction.
*H3*: Hopelessness mediates the relationship between active aging and depressive symptoms.
*H4*: Hopelessness mediates the relationship between active aging and life satisfaction.

## 2 Materials and methods

### 2.1 Participants

Data were collected using a panel provided by a professional research company, dataSpring. An online survey was conducted with 211 older adults in South Korea aged between 65 and 85 years (*M* = 69.14, SD = 4.00). All panel members were Korean and were registered with the survey company. Once the study topic was disclosed, willing participants voluntarily accessed the survey system and completed the questionnaire. Eligibility was restricted to individuals aged 65–85 years, and efforts were made to balance gender distribution. Regarding age distribution, although a relatively even representation across individuals in their 60s, 70s, and 80s would have been ideal, there were practical limitations due to the relatively small number of panel members aged 80 years and older in the online panel. As a result, participants in their 60s constituted the largest proportion of the sample. During the survey process, participants were first informed that the study focused on mental health in later life through a recruitment notice for the survey. They were also provided with detailed information regarding voluntary participation and research ethics. Only those who voluntarily provided informed consent were allowed to complete the survey. This study was approved by the Institutional Review Board (IRB) of the authors' affiliated institution.

### 2.2 Measures

#### 2.2.1 Active aging

To measure active participation among older adults, the “Participation” subscale of the Active Aging Scale developed by Ryu et al. (2014) was used. The original scale, based on the World Health Organization (2002) framework, includes three domains—Participation, Health, and Security—to capture engagment, physical well being, and environmental support, respectively. In the present study, the focus was placed exclusively on the Participation domain to assess intentional and proactive activity, rather than physical or environmental conditions. The full scale comprises seven subdomains: self-development activity, religion activity, daily activity, social interaction, family support activity, voluntary activity, and leisure activity. Considering the Korean context, participation in religion and voluntary activities is relatively infrequent, and daily activities and family support are largely routine; therefore, these subdomains were excluded. The Participation construct thus includes self-development activity, leisure activity, and social interaction, capturing the personal, social, and relational dimensions of meaningful participation. This subscale consists of 14 items, including “I engage in activities that utilize my abilities,” “I participate in programs at cultural centers or welfare facilities (e.g., meditation, singing classes),” and “I attend social gatherings.” Responses are rated on a 5-point Likert scale, with higher average scores indicating greater levels of active participation in later life (α = 0.918, ω = 0.919).

#### 2.2.2 Hopelessness

Hopelessness was assessed using the Beck Hopelessness Scale originally developed by Beck et al. (1974) and translated into Korean by Shin et al. (1990). This scale includes 20 items, such as “My future seems dark to me,” “All I can see ahead of me is unpleasantness rather than pleasantness,” and “I never get what I want, so it's foolish to want anything.” Participants respond to each item with either True (1) or False (0), and the total score reflects the level of hopelessness (α = 0.938, ω = 0.939).

#### 2.2.3 Depressive symptoms

Depressive symptoms was measured using the Korean version of the Patient Health Questionnaire-9 (PHQ-9) originally developed by Spitzer et al. (1999) and translated into Korean by Choi et al. (2007). This 9-item scale asks participants how often they have been bothered by problems such as, “Little interest or pleasure in doing things” and “Feeling bad about yourself or that you are a failure or have let yourself or your family down” over the past 2 weeks. Responses are rated on a 4-point scale (from “Not at all” to “Nearly every day”), and the total score indicates the level of depressive symptoms (α = 0.894, ω = 0.901).

#### 2.2.4 Life satisfaction

Life satisfaction was measured using the Satisfaction With Life Scale (SWLS), developed by Diener et al. (1985) and translated into Korean by Zho and Cha (1998). This scale includes five items assessing overall life satisfaction, such as “In most ways, my life is close to my ideal” and “I am satisfied with my life.” Responses are rated on a 5-point scale, and the mean score is calculated, with higher scores indicating greater life satisfaction (α = 0.884, ω = 0.890).

#### 2.2.5 Demographic variables

Gender, age, subjective health, education level, and economic status were reported (Table 1). All demographic information was collected using single-item measures. Gender was coded as a binary variable (0 = male, 1 = female). Pearson correlations involving gender are therefore equivalent to point-biserial correlations.

**Table 1:** Demographic Characteristics.

Variables	Item	*N*	Percentage
Gender	Male	106	50.2
	Female	105	49.8
Age	60s	134	63.5
	70s	69	32.7
	80s	8	3.8
Subjective health	Very bad	5	2.4
	Bad	34	16.1
	Normal	97	46
	Good	67	31.8
	Very good	8	3.8
Education level	Elementary school	2	0.9
	Middle school	11	5.2
	High school	64	30.3
	University	112	53.1
	Graduate school	22	10.4
Economic status	Low	21	10
	Worse than average	65	30.8
	Average	96	45.5
	Better than average	29	13.7
	High	0	0

### 2.3 Data analysis

The collected data were analyzed using SPSS Statistics version 26.0. The main analytical procedures were as follows. First, descriptive statistics and correlation analyses were conducted to examine the participants' basic characteristics and the relationships between the main variables. Cronbach's α coefficients were calculated for each scale to assess internal consistency reliability. Second, the normality of the data was assessed by examining the skewness and kurtosis of the variables. Simple regression analyses were conducted to check for multicollinearity among the variables. In these analyses, depressive symptoms and life satisfaction were entered as outcome variables, whereas all other variables were included as predictors. Tolerance and Variance Inflation Factor (VIF) values were examined to assess multicollinearity. Third, the mediating effect of hopelessness on the relationship between active aging and both depressive symptoms and life satisfaction was tested using Model 4 of the PROCESS Macro version 13 developed by Hayes (2013). The significance of the indirect effects was assessed using the bootstrapping method with 5,000 resamples. Mediation was considered significant if the 95% confidence interval for the indirect effect did not include zero (Li et al., 2018).

## 3 Results

### 3.1 Preliminary analysis

Table 2 presents the results of correlation analyses. The main results revealed that active aging was significantly negatively correlated with depressive symptoms (*r* = −0.55, *p* < 0.01) and positively correlated with life satisfaction (*r* = 0.72, *p* < 0.01), thus supporting Hypotheses 1 and 2. This indicates that older adults with more active lifestyles tend to report less depressive symptoms and better life satisfaction. Hopelessness, the mediating variable, was significantly negatively correlated with active aging (*r* = −0.73, *p* < 0.01) and life satisfaction (*r* = −0.70, *p* < 0.01), and positively correlated with depressive symptoms (*r* = 0.64, *p* < 0.01). These results indicate that active aging is associated with lower hopelessness, which, in turn, is related to better mental health outcomes. Notably, while some variables exhibited strong correlations (*r* > 0.70), they remain conceptually distinct, representing proactive engagement vs. cognitive outlook, respectively. Nevertheless, the magnitude of these associations should be interpreted with caution, as they may be partially influenced by common method bias inherent in self-reported data.

**Table 2:** Correlations and descriptive statistics (*N* = 211).

Variables	1	2	3	4	5	6	7	8	9
1. Active aging	1								
2. Hopelessness	−0.73^**^	1							
3. Depressive symptoms	−0.55^**^	0.64^**^	1						
4. Life satisfaction	0.72^**^	−0.70^**^	−0.54^**^	1					
5. Subjective health	0.40^**^	−0.38^**^	−0.42^**^	0.46^**^	1				
6. Education level	0.24^**^	−0.17^*^	−0.10	0.20^**^	0.13	1			
7. Economic status	0.47^**^	−0.47^**^	−0.41^**^	0.51^**^	0.47^**^	0.33^**^	1		
8. Gender	−0.01	−0.08	−0.04	0.01	0.03	−0.33^**^	−0.09	1	
9. Age	−0.05	−0.01	−0.06	−0.02	−0.01	−0.08	0.01	−0.06	1
Mean	3.24	6.79	13.71	2.91	3.18	3.67	2.63	1.50	69.14
SD	0.74	6.27	5.11	0.87	0.83	0.77	0.84	0.50	4.00
Skewness	−0.52	0.66	1.44	−0.35	−0.21	−0.55	−0.22	0.01	1.54
Kurtosis	0.56	−0.85	1.97	−0.47	−0.02	0.70	−0.49	−2.02	2.53

^*^p < 0.05, ^**^ p < 0.01.

In addition, correlations between demographic and main variables were examined. Gender and age were not significantly correlated with the main variables. However, subjective health, education level, and economic status were also significantly associated (Table 2). Specifically, better subjective health correlated with higher active aging (*r* = 0.40, *p* < 0.01), lower hopelessness and depressive symptoms (*r* = −0.38 and −0.42, respectively, *p* < 0.01), and higher life satisfaction (*r* = 0.46, *p* < 0.01). Similarly, higher education level was associated with greater active aging (*r* = 0.24, *p* < 0.01), lower hopelessness (*r* = −0.17, *p* < 0.05), and higher life satisfaction (*r* = 0.20, *p* < 0.01). However, education level was not significantly correlated with depressive symptoms (*r* = −0.10, *p* = 0.156). Economic status was positively correlated with active aging (*r* = 0.47, *p* < 0.01), negatively correlated with hopelessness and depressive symptoms (*r* = −0.47 and −0.41, respectively, *p* < 0.01), and positively correlated with life satisfaction (*r* = 0.51, *p* < 0.01). Based on these findings, subjective health, education level, and economic status were included as control variables in the subsequent mediation analyses.

To assess multicollinearity, linear regression analyses were conducted with depressive symptoms and life satisfaction as outcomes. Predictors included active aging, hopelessness, sex, age, subjective health, education, and economic status. Tolerance values ranged from 0.43 to 0.98, and VIF values ranged from 1.03 to 2.34, indicating no multicollinearity among the predictors.

Furthermore, before testing the structural model, a Confirmatory Factor Analysis (CFA) was performed to evaluate the validity of the measurement model. The model fit indices were as follows: χ2 = 2088 (df = 1074, *p* < 0.001), χ2/df =1.94, RMSEA = 0.067, SRMR = 0.066, CFI = 0.836, TLI = 0.827. The absolute fit indices (RMSEA, SRMR) and the normed chi-square (χ2/df) all met the recommended criteria (RMSEA < 0.08, χ2/df < 3.0), indicating an acceptable fit. Although CFI and TLI were slightly below the 0.90 threshold, this is likely due to the high degrees of freedom (1,074) and the binary nature (0/1) of Hopelessness scales used in this study, which tend to produce conservative incremental fit estimates (Bentler and Bonett, 1980; Gefen et al., 2000). All factor loadings were statistically significant (*p* < 0.001), confirming the model was deemed appropriate for proceeding with further structural analysis (Table 3).

**Table 3:** Confirmatory factor analysis and reliability of the measurement scales.

Factor	CFI	TLI	RMSEA	SRMR	χ^2^(df)	α	ω
Active aging	0.826	0.794	0.126	0.069	337 (77)	0.918	0.919
Hopelessness	0.894	0.881	0.080	0.053	401 (107)	0.938	0.939
Depressive symptoms	0.933	0.910	0.106	0.047	91.6 (27)	0.894	0.901
Life satisfaction	0.991	0.982	0.072	0.016	10.5 (5)	0.884	0.890
Total	0.836	0.827	0.067	0.066	2,088 (1,074)	-	-

The RMSEA values for Active Aging and Depressive Symptoms were relatively high, which may be attributed to the small sample size (*N* = 211). In models with small degrees of freedom and limited sample sizes, RMSEA can be artificially inflated and should be interpreted with caution (Kenny et al., 2015). However, the SRMR values for these scales were 0.069 and 0.047, respectively, meeting the goodness-of-fit criterion of < 0.08 (Hu and Bentler, 1999). Additionally, the χ^2^/df ratios were 4.37 and 3.39, which are within the acceptable range of < 5.0 (Carmines and McIver, 1981; Wheaton et al., 1977). Considering these indices alongside the high internal consistency (ω >0.90), all original items were retained to maintain the theoretical integrity of the constructs.

### 3.2 Mediation effects

#### 3.2.1 The mediating effect of hopelessness on the relationship between active aging and depressive symptoms

First, the mediating effect of hopelessness on the relationship between active aging and depressive symptoms was confirmed, thus supporting Hypothesis 3 (Table 4). Active aging was significantly negatively associated with hopelessness (*B* = −5.51, *p* < 0.001), and hopelessness was significantly positively associated with depressive symptoms (*B* = 0.37, *p* < 0.001). However, the direct effect of active aging on depressive symptoms after controlling for hopelessness was not statistically significant (*B* = −0.95, *p* = 0.083), while the total effect without controlling for hopelessness was significant (*B* = −2.99, *p* < 0.001). The mediation model explained a substantial proportion of the variance in hopelessness (*R*^2^ = 0.56) and depressive symptoms (*R*^2^ = 0.46). The significance of the indirect effects was confirmed using bootstrapping. The 95% confidence interval for the indirect effect did not include zero, indicating a statistically significant mediation effect [*B* = −2.05, BootSE = 0.46, 95% CI (−3.00, −1.16)].

**Table 4:** The mediating effect of hopelessness on the relationship between active aging and depressive symptoms.

Pathway	*B*	SE	*t*	95%CI	
				Lower	Upper
Active aging → hopelessness	−5.51	0.46	−11.88^***^	−6.42	−4.60
Subjective health	−0.44	0.41	−1.09	−1.25	0.36
Education level	0.36	0.40	0.89	−0.44	1.15
Economic status	−1.12	0.43	−2.58^*^	−1.97	−0.26
Hopelessness → depressive symptoms	0.37	0.06	5.91^***^	0.25	0.50
(Direct effect) Active aging → depressive symptoms	−0.95	0.54	−1.74	−2.02	0.12
Subjective health	−0.98	0.37	−2.66^**^	−1.71	−0.25
Education level	0.39	0.36	1.07	−0.33	1.11
Economic status	−0.48	0.40	−1.20	−1.26	0.31
(Total effect) Active aging → depressive symptoms	−2.99	0.45	−6.63^***^	−3.88	−2.10
Subjective health	−1.14	0.40	−2.89^**^	−1.92	−0.36
Education level	0.52	0.39	1.33	−0.25	1.29
Economic status	−0.89	0.42	−2.12^*^	−1.72	−0.06
(Indirect effect) Active aging → hopelessness → depressive symptoms	−2.05	0.46 (Boot)	-	−3.00	−1.16

^*^p < 0.05, ^**^p < 0.01, ^***^p < 0.001.

Control Variables: subjective health, education level, economic status.

#### 3.2.2 The mediating effect of hopelessness on the relationship between active aging and life satisfaction

Second, the mediating effect of hopelessness on the relationship between active aging and life satisfaction was confirmed, thus supporting Hypothesis 4 (Table 5). Active aging was significantly negatively associated with hopelessness (*B* = −5.51, *p* < 0.001), and hopelessness was significantly negatively associated with life satisfaction (*B* = −0.04, *p* < 0.001). The direct effect of active aging on life satisfaction after controlling for hopelessness remained significant (*B* = 0.46, *p* < 0.001), and the total effect without controlling for hopelessness was also significant (*B* = 0.70, *p* < 0.01). The mediation model explained a substantial proportion of the variance in hopelessness (*R*^2^ = 0.56) and life satisfaction (*R*^2^ = 0.62). The significance of the indirect effect was verified using bootstrapping. The 95% confidence interval for the indirect effect did not include zero, indicating a statistically significant mediating effect [*B* = 0.24, BootSE = 0.06, 95% CI (0.13, 0.36)].

**Table 5:** The mediating effect of hopelessness on the relationship between active aging and life satisfaction.

Pathway	*B*	SE	*t*	95%CI	
				Lower	Upper
Active aging → hopelessness	−5.51	0.46	−11.88^***^	−6.42	−4.60
Subjective health	−0.44	0.41	−1.09	−1.25	0.36
Education level	0.36	0.40	0.89	−0.44	1.15
Economic status	−1.12	0.43	−2.58^*^	−1.97	−0.26
Hopelessness → Life Satisfaction	−0.04	0.01	−4.86^***^	−0.06	−0.03
(Direct Effect) Active Aging → life satisfaction	0.46	0.08	5.91^***^	0.30	0.61
Subjective health	0.14	0.05	2.73^**^	0.04	0.25
Education level	−0.004	0.05	−0.07	−0.11	0.10
Economic status	0.12	0.06	2.17^*^	0.01	0.23
(Total effect) Active aging → life satisfaction	0.70	0.06	11.10^***^	0.57	0.82
Subjective health	0.16	0.06	2.95^**^	0.05	0.27
Education level	−0.02	0.05	−0.36	−0.13	0.09
Economic status	0.17	0.06	2.92^**^	0.06	0.29
(Indirect effect) Active aging → hopelessness → life satisfaction	0.24	0.06 (Boot)	-	0.13	0.36

^*^p < 0.05, ^**^p < 0.01, ^***^p < 0.001.

Control variables: subjective health, education level, economic status.

## 4 Discussion

### 4.1 Main findings

This study examined whether an active life in old age links to better mental health, specifically lower depressive symptoms and higher life satisfaction, and whether this relationship is mediated by perceived hopelessness. First, active aging was significantly associated with both lower depressive symptoms and higher life satisfaction. These findings are consistent with prior research (Galli et al., 2016; Kahneman and Krueger, 2006; Li et al., 2018; Litwin, 2000) and emphasize the benefits of meaningful engagement in later life. As older adults face life transitions, such as retirement and reduced social roles (Shanas, 1962), active living can help counter emotional vulnerability and maintain a sense of identity. Second, hopelessness mediated the relationship between active aging and depressive symptoms Since hopelessness is a known predictor of depression (Abramson et al., 1989), an active lifestyle tended to co-occur with less pessimistic thinking and lower emotional exhaustion, which often increase with age (Coudin and Lima, 2011). Third, hopelessness mediated the link between active aging and life satisfaction. This indicates that an active life can foster a more hopeful and positive outlook, which in turn enhances overall well being. Notably, the high explanatory power of these models, accounting for 46%−62% of the variance in depressive symptoms and life satisfaction, further underscores the practical significance of these pathways. Overall, the findings highlight the critical role of active aging in promoting mental health by lower hopelessness and offer practical implications for supporting well being in older adults.

### 4.2 Implications

The results of this study have several important implications. First, it contributes to the growing literature on active aging by providing empirical evidence of its link to hopelessness, depressive symptoms, and life satisfaction. As population aging increases worldwide, understanding the benefits of active and meaningful engagement in later life has become increasingly important. These findings support previous research indicating that physical activity, social interaction, and leisure contribute to a better quality of life (Cho and Kim, 2023; Li et al., 2018; Wang et al., 2020) and emphasize the value of maintaining an autonomous, proactive lifestyle in older adulthood. Second, this study advances existing knowledge by identifying hopelessness as a key mediator in the relationship between active aging and mental health. Although many studies have examined the direct effects of activity on well being, fewer have addressed how hopelessness explains this relationship. Given that hopelessness often increases among older adults experiencing physical or cognitive decline, these findings highlight the importance of fostering hope and purpose to support emotional resilience. Third, this study offers practical guidance for individuals navigating the transition into later life. Aging inevitably brings change, but continued engagement and social interaction can help preserve identity, self-efficacy, and psychological health. These results suggest that personal effort to remain active can reduce depressive symptoms and enhance happiness. Finally, this study provides a direction for mental health programs and policy development. Community-based services that promote physical activity, cultural participation, and social connections may enhance older adults' emotional well being. At the policy level, designing welfare systems for older adults to actively encourage participation and reduce isolation is vital. Strengthening local resources can help support mental health and improve the quality of life in an aging society. Such approaches could help minimize the growing issue of social isolation in modern society and contribute to improving mental health and social connectedness among older adults.

### 4.3 Limitations and suggestions for future studies

This study has several limitations that provide directions for future research. First, it used a cross-sectional survey method, which limits causal interpretation. While active aging is associated with lower hopelessness and better mental health, the reverse may also be true; individuals with lower hopelessness may be more likely to engage in activities. Besides, as all variables were assessed through self-report measures within a single survey, the potential for common method bias cannot be entirely ruled out. Thus, future research should adopt experimental or longitudinal designs to better establish causality and examine long-term effects. Second, this study treated older adults as a homogeneous group without accounting for individual differences. However, some individuals may prefer a quiet, introspective lifestyle, whereas others seek social or physical engagement. Moreover, it is possible that the relationship between active aging and mental health could be confounded by underlying factors such as innate sociability, perceived social support, baseline stress level, or the presence of chronic illnesses, which may independently influence both participation levels and psychological well being. In addition, as this study was conducted as an online survey, the potential for digital access bias should be considered, as it may reflect characteristics of older adults who are more comfortable using digital devices. Accordingly, future studies should examine how psychological traits and social backgrounds affect this relationship. Third, the definition of “older adulthood” could be refined. Although 65 is commonly used as the threshold for old age, many in this group remained active and youthful. Previous studies have proposed distinguishing between early-old (65–74 years) and late-old (75+ years) adults because they tend to show meaningful differences in physical, cognitive, and psychological functioning (De Beni et al., 2013; von Humboldt and Leal, 2017). Research has also shown that late-old individuals tend to experience more psychological and physical difficulties than their younger counterparts (Lee, 1999; Lim and Kim, 2012). Therefore, future studies that consider these realistic aspects of the aged population and compare individuals aged approximately 75 years could yield more specific results. Fourth, while this study utilized depressive symptoms and life satisfaction as primary mental health indicators, future research could incorporate a broader range of variables—such as psychological well being, happiness, anxiety, and stress—to provide a more comprehensive understanding of the role of active aging in promoting mental health among older adults. Finally, future research should consider the cultural context. This study was based on a sample of Korean older adults, whose living arrangements and social expectations may differ from those in other cultures. For instance, many Korean seniors still live with relatives or maintain strong family-centered support systems, which could influence the impact of active aging on mental health. In collectivist societies, relational activities may yield stronger emotional benefits, whereas in individualistic cultures, solitary or self-focused activities may be more meaningful. Therefore, future research should explore how the definitions and effects of active aging vary across cultural backgrounds, rather than assuming a universal model.

## 5 Conclusion

As society enters an era of aging, the desire for a better quality of life and mental health has grown. As lifespans increase, individuals may experience emotional difficulties while adapting to these changes, making continuous effort necessary to maintain life satisfaction. Active aging supports healthy adaptation to life-cycle changes through individuals' voluntary efforts, complemented by appropriate social policies. Through these personal and societal efforts, older adults can maintain a higher quality of life, making happiness later in life more attainable.

## Data Availability

The data sets for this study can be found in the Open Science Framework (OSF) at: https://osf.io/zhabf/?view_only=6118bbd64adf42f39d1eb02c761c3bb8.
